# Supplementary material for: Quantitative proteomics identifies PTP1B as modulator of B cell antigen receptor signaling
Source: Life Sci Alliance. 2021 Sep 15;4(11):e202101084. doi: 10.26508/lsa.202101084 (PMC8473724; doi:10.26508/lsa.202101084)

Source files to Fig 3A-C

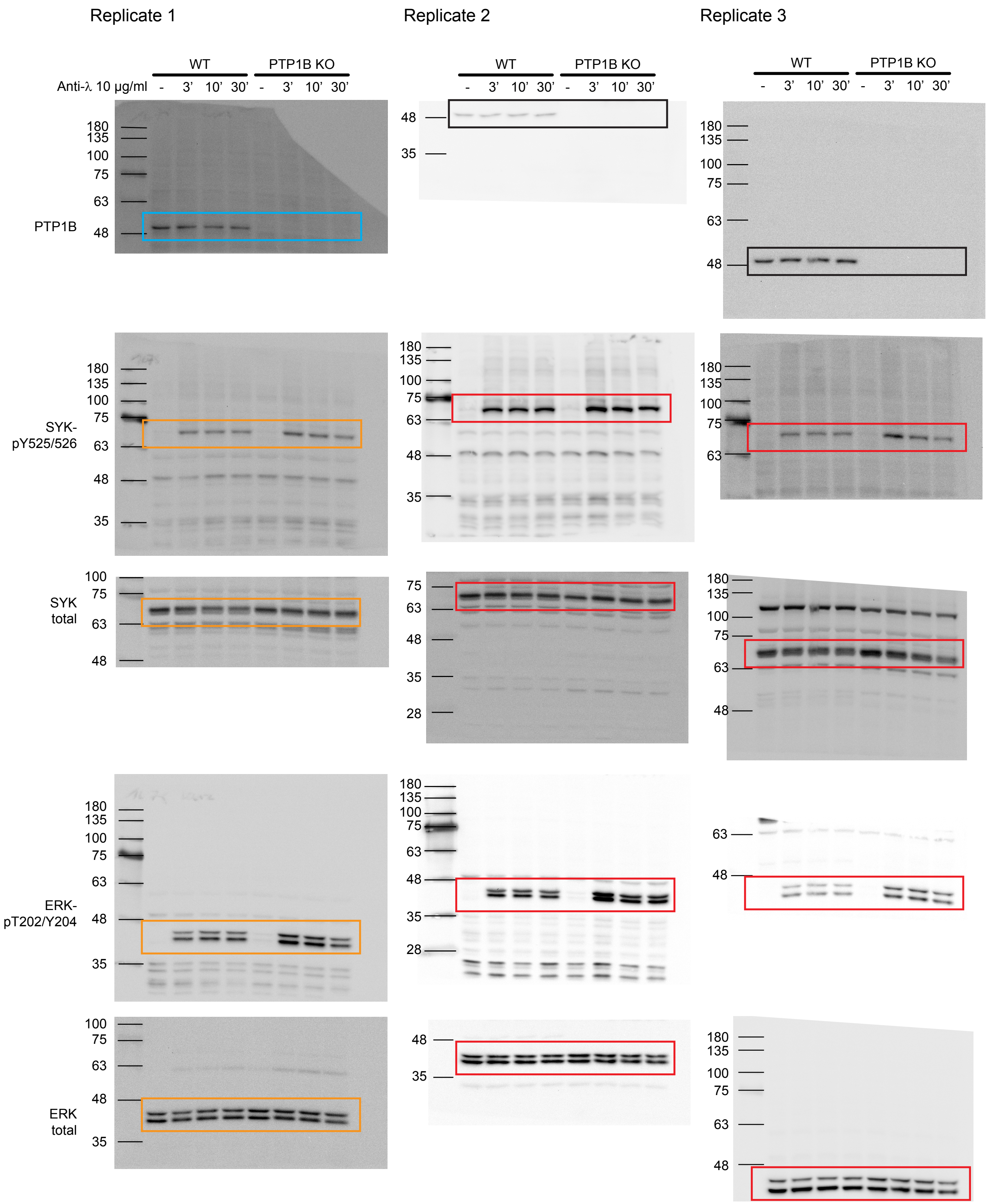

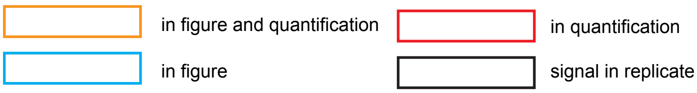

Source files to Fig 3D-H

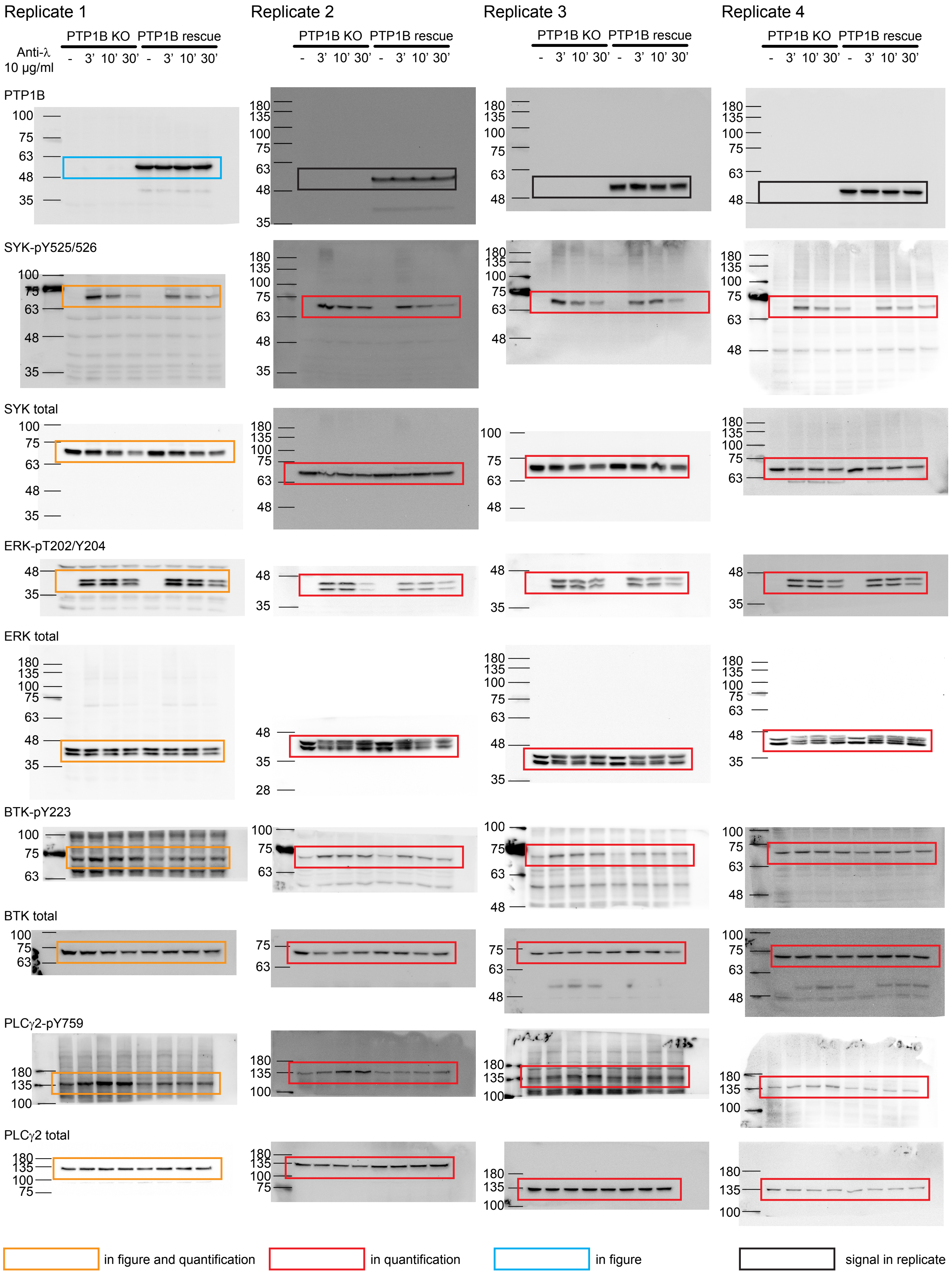

Supplement: Supplementary file 9 [file LSA-2021-01084_SdataF3.pdf]
