# Supplementary figures and images for: Quantitative proteomics identifies PTP1B as modulator of B cell antigen receptor signaling
Source: Life Sci Alliance. 2021 Sep 15;4(11):e202101084. doi: 10.26508/lsa.202101084 (PMC8473724; doi:10.26508/lsa.202101084)

Source files to Fig 5A

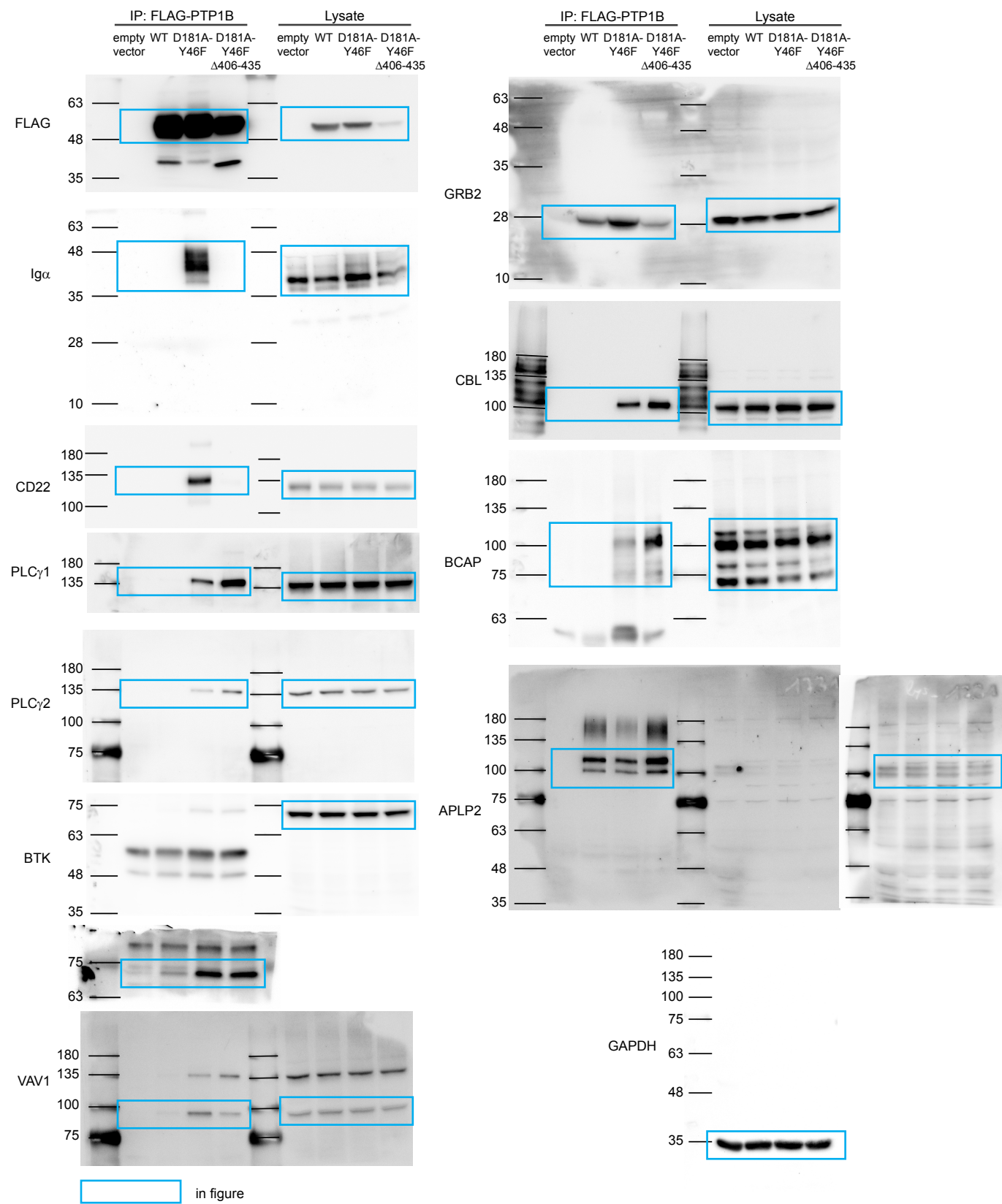

Source files to Fig 5B

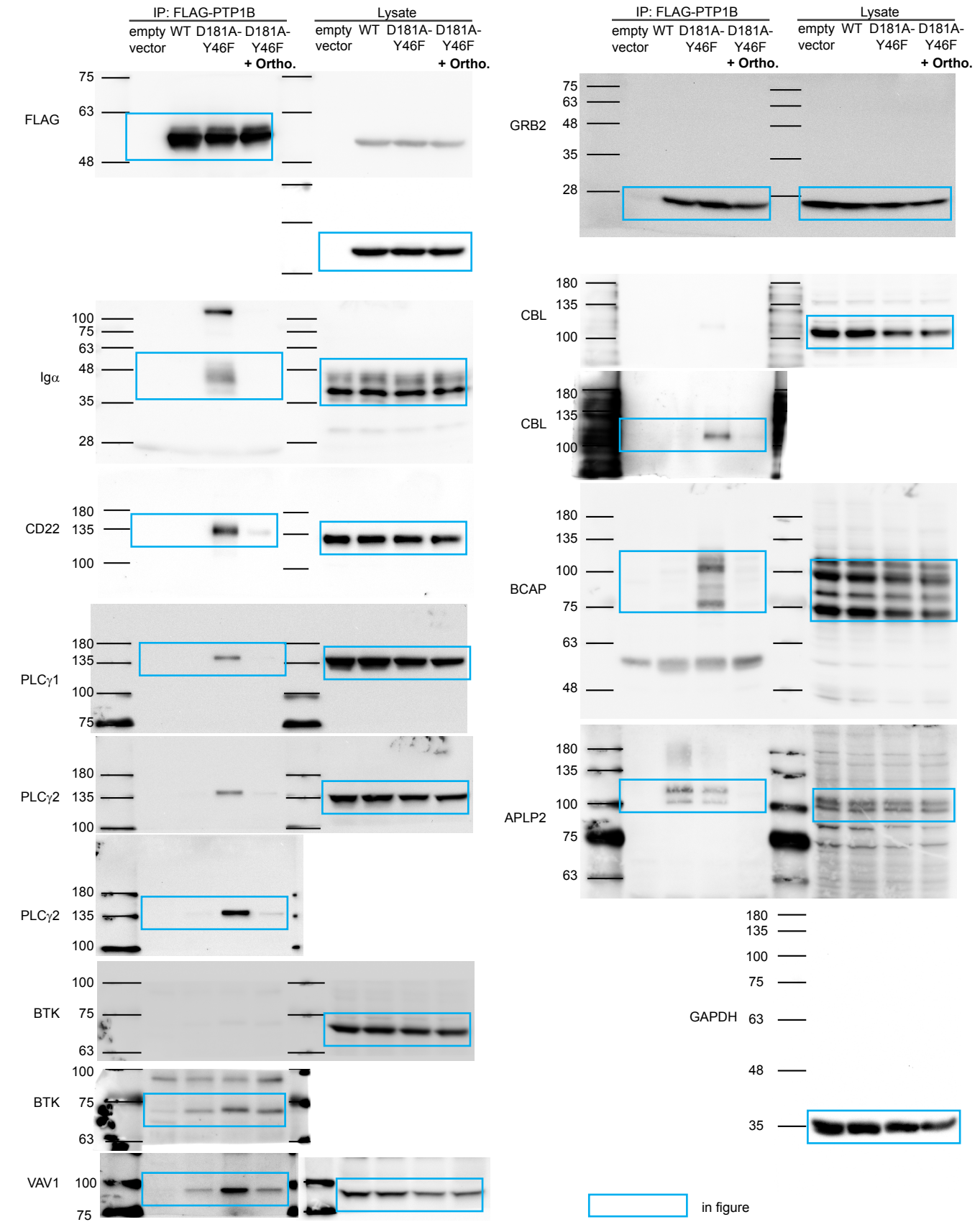

Supplement: Supplementary file 11 [file LSA-2021-01084_SdataF5.pdf]
